# Development of the ehive Digital Health App: Protocol for a Centralized Research Platform

**DOI:** 10.2196/49204

**Published:** 2023-11-16

**Authors:** Robert P Hirten, Matteo Danieletto, Kyle Landell, Micol Zweig, Eddye Golden, Georgy Orlov, Jovita Rodrigues, Eugenia Alleva, Ipek Ensari, Erwin Bottinger, Girish N Nadkarni, Thomas J Fuchs, Zahi A Fayad

**Affiliations:** 1 Henry D Janowitz Division of Gastroenterology Icahn School of Medicine at Mount Sinai New York, NY United States; 2 Hasso Plattner Institute for Digital Health at Mount Sinai New York, NY United States; 3 Windreich Department of Artificial Intelligence and Human Health Icahn School of Medicine at Mount Sinai New York, NY United States; 4 Charles Bronfman Institute for Personalized Medicine, Icahn School of Medicine at Mount Sinai New York, NY United States; 5 BioMedical Engineering and Imaging Institute Icahn School of Medicine at Mount Sinai New York, NY United States; 6 Department of Diagnostic, Molecular and Interventional Radiology Icahn School of Medicine at Mount Sinai New York, NY United States

**Keywords:** digital health, wearable devices, research platform, eHive, smartphone, wearables, digital technologies, digital study, multimodal data collection, framework, development, centralized digital research platform, ehive app, mobile phone

## Abstract

**Background:**

The increasing use of smartphones, wearables, and connected devices has enabled the increasing application of digital technologies for research. Remote digital study platforms comprise a patient-interfacing digital application that enables multimodal data collection from a mobile app and connected sources. They offer an opportunity to recruit at scale, acquire data longitudinally at a high frequency, and engage study participants at any time of the day in any place. Few published descriptions of centralized digital research platforms provide a framework for their development.

**Objective:**

This study aims to serve as a road map for those seeking to develop a centralized digital research platform. We describe the technical and functional aspects of the ehive app, the centralized digital research platform of the Hasso Plattner Institute for Digital Health at Mount Sinai Hospital, New York, New York. We then provide information about ongoing studies hosted on ehive, including usership statistics and data infrastructure. Finally, we discuss our experience with ehive in the broader context of the current landscape of digital health research platforms.

**Methods:**

The ehive app is a multifaceted and patient-facing central digital research platform that permits the collection of e-consent for digital health studies. An overview of its development, its e-consent process, and the tools it uses for participant recruitment and retention are provided. Data integration with the platform and the infrastructure supporting its operations are discussed; furthermore, a description of its participant- and researcher-facing dashboard interfaces and the e-consent architecture is provided.

**Results:**

The ehive platform was launched in 2020 and has successfully hosted 8 studies, namely 6 observational studies and 2 clinical trials. Approximately 1484 participants downloaded the app across 36 states in the United States. The use of recruitment methods such as bulk messaging through the EPIC electronic health records and standard email portals enables broad recruitment. Light-touch engagement methods, used in an automated fashion through the platform, maintain high degrees of engagement and retention. The ehive platform demonstrates the successful deployment of a central digital research platform that can be modified across study designs.

**Conclusions:**

Centralized digital research platforms such as ehive provide a novel tool that allows investigators to expand their research beyond their institution, engage in large-scale longitudinal studies, and combine multimodal data streams. The ehive platform serves as a model for groups seeking to develop similar digital health research programs.

**International Registered Report Identifier (IRRID):**

DERR1-10.2196/49204

## Introduction

### Background

The increasing use of smartphones has provided an opportunity for the growth of mobile health apps for studying health and disease. These apps enable engagement with participants throughout the day, remotely, and at a high frequency. They allow for the collection of patient-reported outcomes measures (PROMs) through surveys, the assessment of social media use, website tracking, and the examination of patterns of phone use [1]. Similarly, wearable and connected devices for ambient sensing are increasingly accepted and used in the United States [2]. Commercial and custom devices can collect physiological data from users continuously and without active input.

Digital technologies are increasingly used to help overcome the limitations of traditional research, such as limitations to recruiting sufficiently large and diverse cohorts and tracking day-to-day changes in participant conditions [3,4]. Remote digital study platforms represent a subset of digital platforms comprising a patient-interfacing digital application that enables multimodal data collection from a mobile app and connected sources. They offer an opportunity to recruit at scale, acquire data longitudinally at a high frequency, and engage study participants at any time of the day in any place [5,6]. These multimodal platforms collect PROMs, provide study content such as videos or other engagement tasks, and collect objective environmental and connected device data. They can enable the electronic onboarding of participants through an e-consent process and establish individual baselines that allow for the construction and forecasting of individual patient trajectories [7].

Many app-based studies and platforms often suffer from low rates of retention and adherence [8-11]. Factors such as perceived utility, value, convenience, and usability have been identified as barriers to use [12]. However, a centralized and agile digital research platform offers an opportunity to improve engagement and mitigate many factors that drive low adherence rates. Through the concerted development and expansion of a central modifiable research application, usability and engagement tools can be optimized across the platform. This mitigates the need to develop single-use applications, enabling the successful growth of digital research across disciplines. The ehive studies platform is the centralized digital research application of the Mount Sinai Health System (MSHS) in New York. It is a proof-of-concept platform that incorporates the elements and features most necessary across studies, uses recruitment and retention features, outlines data collection elements, and assures patient privacy and safety. It serves as a potential model for other health systems and organizations seeking to develop centralized digital research programs.

### Overview

Here, we describe the ehive research platform and its features to provide a road map for the development of similar infrastructure. In the following sections, we first describe the technical aspects and functionalities of the ehive platform, including participant consenting procedures and privacy protection. Then, we provide information about ongoing studies hosted on ehive, including usership statistics and data infrastructure. Finally, we discuss our experience with ehive in the broader context of the current landscape of digital health research platforms.

## Methods

### Digital Platform Development

The MSHS (New York, New York) includes the Icahn School of Medicine at Mount Sinai and 8 hospitals in the New York metropolitan area. It comprises the largest health system in New York City, with one of the most diverse patient populations in the United States [13]. The Hasso Plattner Institute for Digital Health at Mount Sinai (HPI.MS) was founded in 2019 and represents an international academic collaboration between the Hasso Plattner Institute for Digital Engineering in Potsdam, Germany, and the MSHS in New York City, United States [14]. The COVID-19 pandemic highlighted the need for a digital alternative to the in-person studies conducted by the MSHS, which was seen as an imperative future direction. To meet this need, HPI.MS launched the ehive digital research platform in early 2020 with the initial goal of creating a platform to support multiple studies and study types. The initial design of ehive drew upon the prior experiences of the digital health and clinical research faculty, who were charged with its development. The ehive platform is overseen by a program within HPI.MS called the Digital Discovery Program (DDP [15]). The DDP is responsible for maintaining ehive and determining which studies will use the platform and how the platform will be modified and evolve over time. Although the DDP determines the short- and long-term goals of the app, input is solicited from researchers using the app and study participants. Study-specific modifications are undertaken during the planning and development phase of each study, which can be applied across the platform. Any unsolicited feedback from the participants using the app is evaluated by the DDP and incorporated into future modifications.

This multimodal digital platform can be used on both iOS (Apple Inc) and Android (Google LLC) mobile devices and is designed to support multiple independent research studies via the collection of remote consent and PROMs and integration of data from commercial and custom wearable devices, electronic health records (EHRs), and biobanks.

### Recruitment, Retention, and Monitoring

Research participant recruitment and retention is challenging and an important determinant of research success [16]. Digital tools such as email messages, SMS text messages, websites, and smartphone apps are increasingly used to assist with participant recruitment and retention and help eligible participants find potential studies [17]. The ehive platform was developed with unique capabilities to assist investigators with study recruitment, including bulk messaging through the EPIC EHR system and standard email portals. The lists of potential study participants meeting the specified inclusion criteria can be generated through a data warehouse system hosted by Mount Sinai. This system collates identified and deidentified data from the EHR, including email addresses. Using this infrastructure, investigators can run queries through the EPIC system to find participants who meet the study eligibility criteria. Through ehive, investigators can send automated recruitment messages to the EPIC MyChart messaging inbox of this cohort. Alternatively, ehive can send automated messages via email using prespecified email distribution lists collected from the data warehouse. These 2 approaches allow for broad engagement with potential participants, enhancing study visibility throughout the health system. This facilitates the recruitment of diverse participant populations, the lack of which is a significant limitation in many digital studies. The ability to engage with individuals across the health system ensures that a large population of patients can become aware of specific studies relevant to them, including patient populations that may be difficult to reach via traditional recruitment methods and across socioeconomic statuses.

Participant retention and engagement are key drivers of data quality and research study success. Over half of the participants in digital studies stop participating after the first week [18]. Frequent light-touch reminders for participants to engage in study activities are often used in digital studies to maintain compliance [12]. The ehive platform was developed with several light-touch features. Smartphone push notifications are generated at customizable frequencies, in customizable language, and at modifiable times of the day to remind participants to complete study tasks and use their connected devices. The app can send modifiable email messages to participants that welcome them to the study, thank them for participating, and remind them to maintain engagement. The ehive app is designed for automated compliance monitoring using customizable engagement thresholds for each study, which generate these customized push notifications or emails for participants or study staff. This permits the passive monitoring of a large number of study participants and initiates re-engagement if compliance drops below the specified thresholds. Depending on the specific study, the app allows participants to tailor their ehive experience based on their daily schedule, providing them the option to customize when they receive notifications and which notifications they receive.

### Research Dashboard

**Figure 1:**
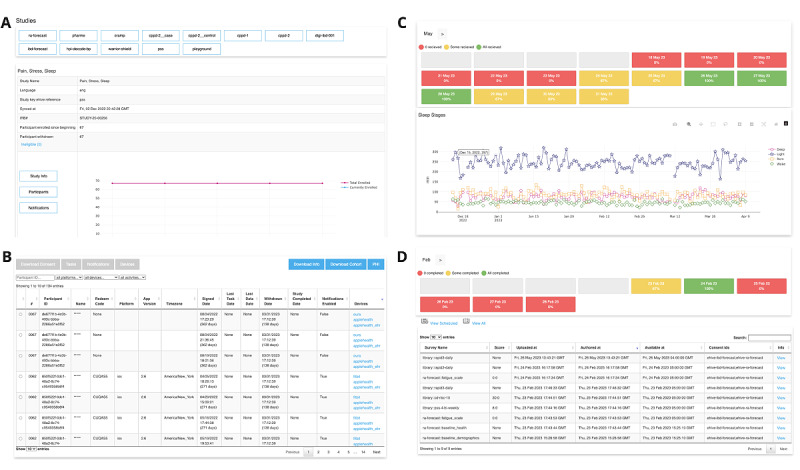
The ehive study dashboard for participant monitoring. (A) Individual studies can be selected to gather study details and visually track enrollment and compliance. (B) Participant information can be visualized for each study, which is provided as an interactive list with participant contact information, unique study IDs, date and time of enrollment, withdrawal date, copies of each signed consent form, and information on patient-reported outcomes measures (PROMs) and wearable data. (C) Wearable device data trends can be visualized for each participant, including sleep and heart rate variability data. (D) Survey data from each participant can be visualized.

A central ehive dashboard is available to monitor participant engagement across studies (Figure 1). The dashboard allows the visualization of ongoing and completed studies and can be tailored to grant specific personnel access to specific studies. Access is granted via invitation, and the dashboard is accessible only through the MSHS virtual private network. The Azure Active Directory manages dashboard log-ins, and role access is managed by an honest broker who has administrative access to the dashboard. The dashboard has multiple features, including an interface for participant and study team communication, a data review feature, and a feature for visualizing data trends based on the information collected from wearable devices and PROMs. Access to each feature can be granted based on the role of the user in each study, as specified in the institutional review board (IRB) approval. This allows research coordinators to actively interact with study participants through custom push notification features or emails, which can be generated within the dashboard. In addition, this allows researchers to interact with the data in a deidentified or an identified fashion based on the study requirements.

Various customizable features allow the study team to change the consent language, task language, and task scheduling as well as to add and remove integrated wearable devices. Customization can be done seamlessly and does not require the release of an app update. This agile solution enhances user experience by allowing researchers to correct errors and provide real-time feature deployment without triggering app updates. Major app updates are available on digital stores (Apple App Store [Apple Inc] and Google Play [Google LLC]), and participants are automatically notified to manually update the app. The ehive team encourages a soft launch of each study to enable the identification and correction of any problems. After the enrollment of the first several participants, study compliance and data quality can be reviewed to enable early modifications of task language or schedules before full-scale enrollment is undertaken. Each data point collected through ehive is time-stamped. All changes to the consent procedure, tasks, or other study components are stored in a change log within GitHub. Using the time stamp associated with study data and the change log, data versions can be tracked and evaluated during the analysis phase.

### Downloading ehive and Providing e-Consent

**Figure 2:**
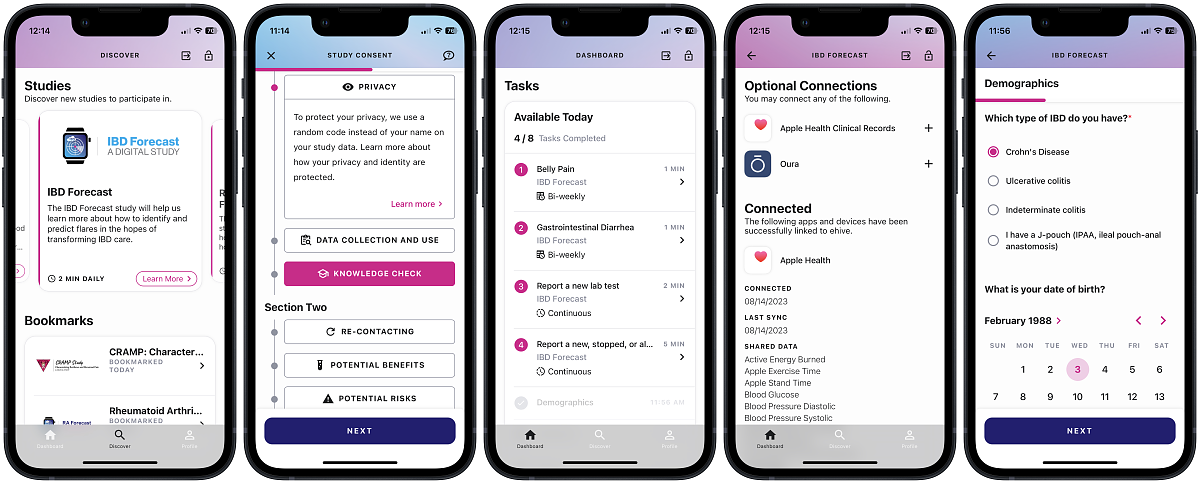
An overview of the major features of the ehive app. Participants can scroll through the available studies, provide consent for studies that interest them and whose eligibility criteria they meet, complete daily tailored study tasks, and share wearable and other health data. IBD: inflammatory bowel disease.

The ehive app can be downloaded free of charge from the Apple App Store and Google Play and is designed for users to remotely onboard themselves (Figure 2). The introduction screen allows individuals to view publicly available studies and publications generated from research conducted on the platform. This interface allows users to explore publicly available studies and join these studies if they meet the eligibility criteria. Non–publicly available studies are not visible in the ehive app unless potential participants are provided with a link and log-in credentials to reveal and unlock the study on their ehive dashboard.

**Figure 3:**
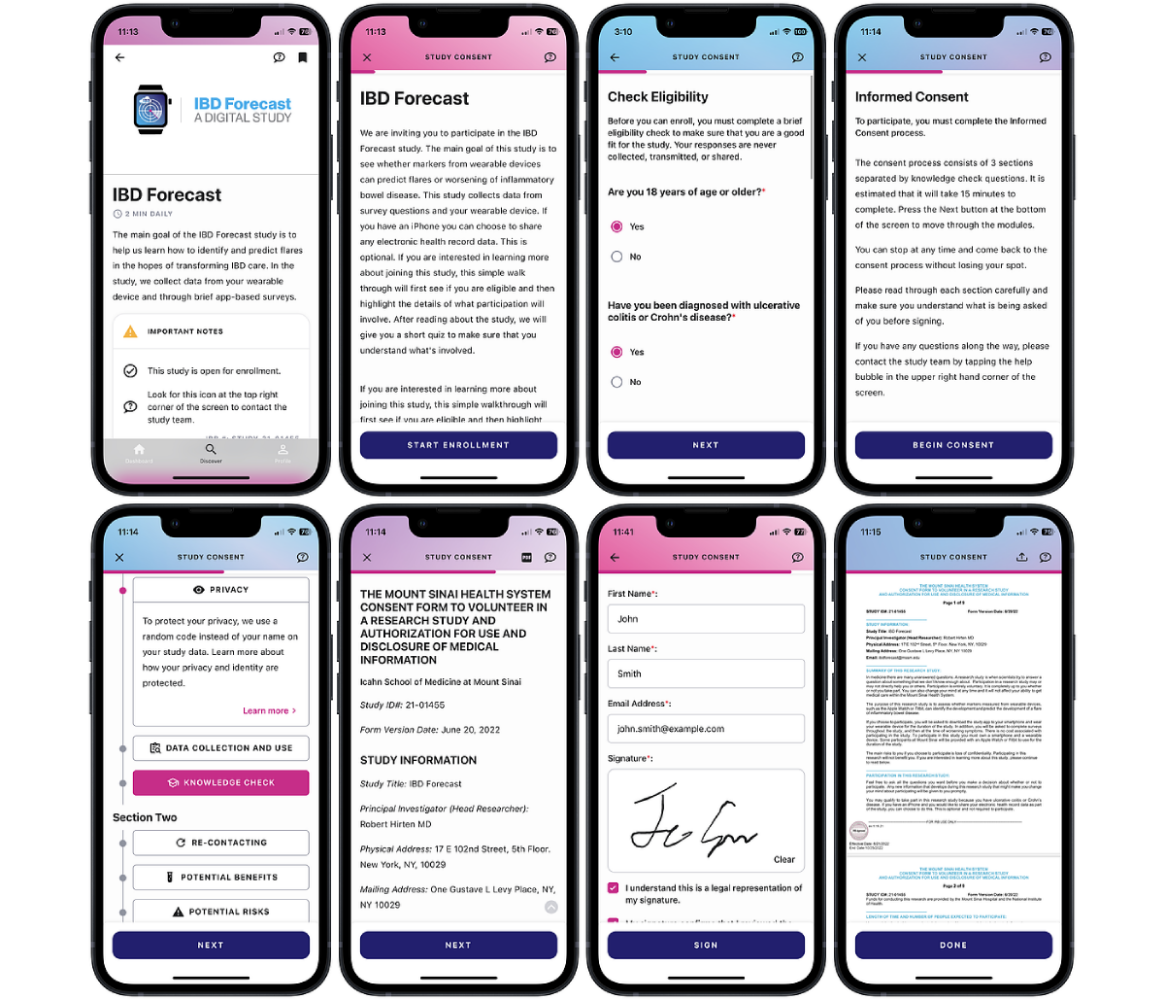
The sample e-consent process in ehive. A brief introduction to the study is provided, followed by participants checking whether they meet the eligibility criteria. If inclusion and exclusion criteria are met, participants are taken through the consent form and required to complete a study comprehension quiz. If this is successful, participants can read through the institutional review board–approved consent form and provide an electronic signature. A pdf version of the signed consent form is generated and available for review. IBD: inflammatory bowel disease.

**Figure 4:**
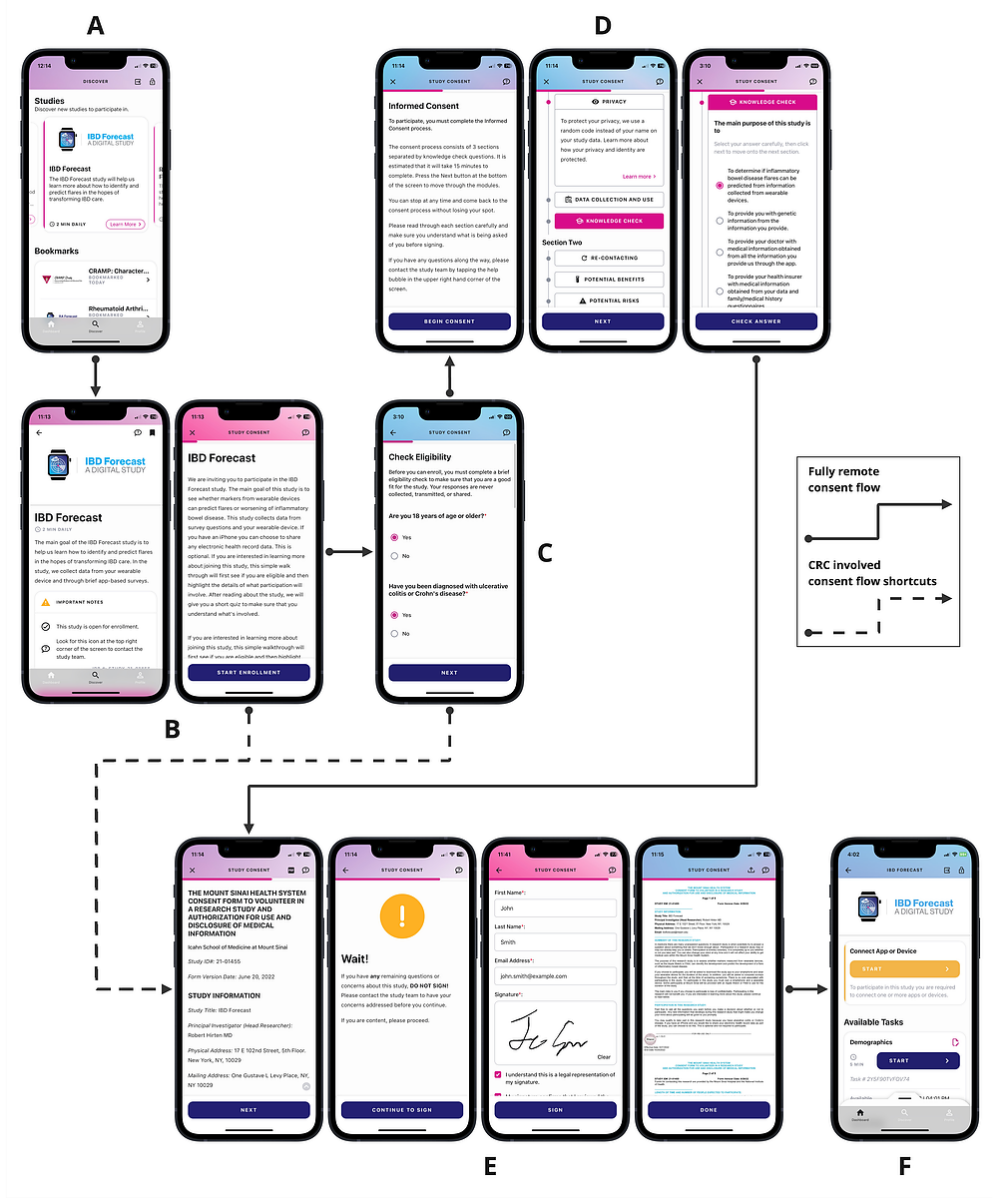
The consent flow for prospective study participants. The consent flow consists of 6 main screen groupings: (A) selection of the study, (B) review of detailed study information, (C) eligibility screening, (D) walkthrough of consent details and knowledge checks, (E) consent document review and signature, and (F) enrolled patient-facing study dashboard. Studies that are fully remote and have no active research coordinator participation include consents using each of these groups. Consent flow is indicated by the solid line in the flow diagram. The consent process of studies using an active research coordinator may skip certain groups if desired by following the dashed line on the flow diagram. CRC: clinical research coordinator; IBD: inflammatory bowel disease.

The consent process is customizable for each study and can include a brief study introduction; an eligibility screener; and, subsequently, an e-consent review and signature process (Figure 3). The eligibility screener verifies that the participant meets the study criteria. This verification of inclusion and exclusion criteria matches user’s answers to questions locally on the phone and does not store this information as study data, as this is done before the study consent process. The subsequent e-consent process incorporates visualizations on the interface to explain concepts, communicate the impacts of the study, and highlight the benefits and risks to participants. This process allows potential participants to read and learn about the study information at their own pace, with the option of contacting the study team with questions at any point. A review of the concepts of e-consent is followed by a series of comprehension questions that assess the participant’s understanding of the key aspects of the study. The e-consent process allows participants to move back and review any material again, as needed. If potential participants fail the study concept quiz 3 times, they are deemed ineligible for the study, and the study is locked to future enrollment. This can be manually reset by the study team if required. Once the study concept quiz is completed by a potential participant, the IRB-approved consent form is available for review and electronic signature. A signed pdf version of the consent form is then generated and stored in the app for future reference. Depending on the study requirements, the e-consent process can be performed either solely by the participant or can involve a study coordinator (Figure 4).

**Figure 5:**
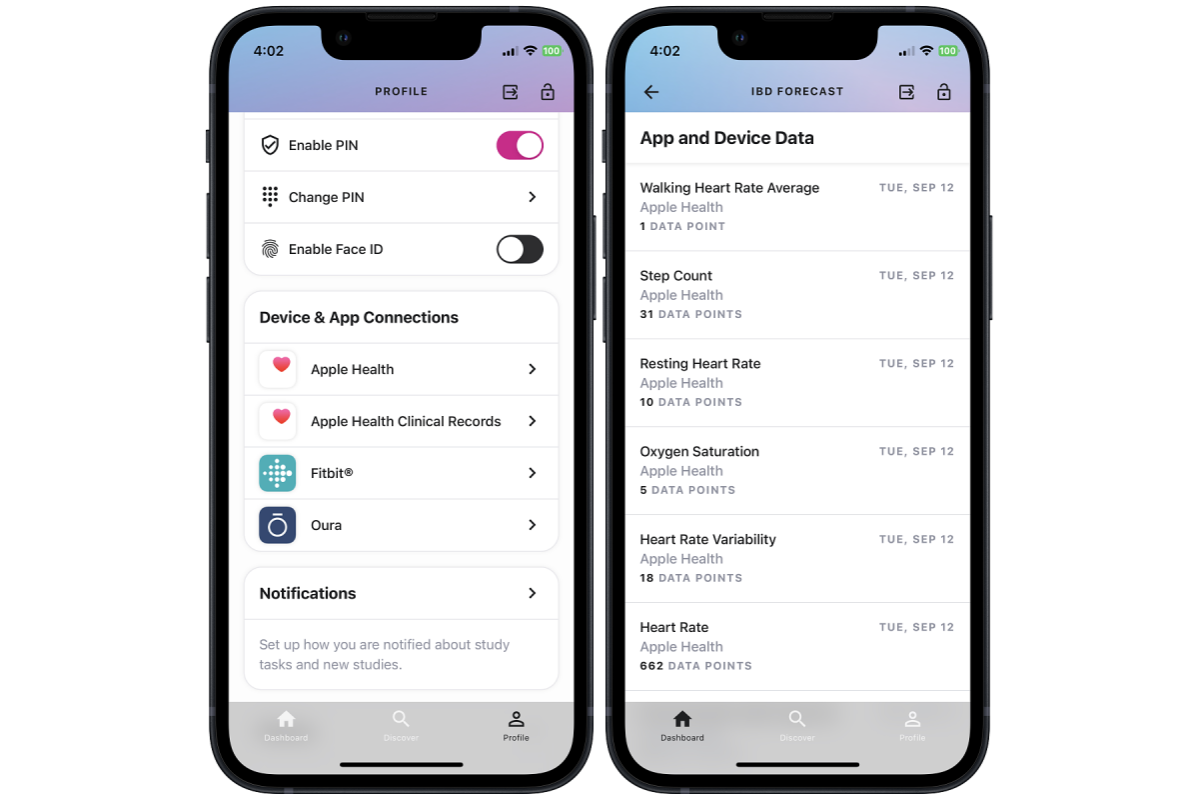
Study integration with external data sources, such as wearable devices. Participants can link connected devices to the ehive app and choose which device data to share with the study team. Participants can verify that app and device data have been shared with the study team. IBD: inflammatory bowel disease.

After the e-consent form is signed, participants can review and select the data streams to be shared with the research team through a data control permission process (Figure 5). Data streams can range from wearable device data to other modalities such as EHR data. If a participant is enrolled in a second study, the app will notify them regarding the need to once again select the data streams to be shared. Permission for wearable data access is provided at the study level, which allows the participant to use the same or additional devices and decide which data to share for each study. This places data-sharing control in the hands of participants and allows them to customize their choice for each study. This permission is modifiable and can be edited or revoked by participants at any time.

### Ethical Considerations

All ehive studies that involve human participants require separate approval from relevant IRBs. Ongoing IRB approval is required for the study to remain active on the platform. Approval is needed for any secondary analyses of research data, for the sharing of data outside the study team, and during the data extraction and analysis period. Each study incorporates its own IRB-approved data-sharing and consent framework. Compensation is individualized for each study. The ehive platform is itself approved by the IRB at the Icahn School of Medicine (STUDY-21-01500) to assure compliance of its consent and data storage approaches with relevant institutional guidelines for the ethical conduct of research. All the data presented in the subsequent sections were approved by the Mount Sinai IRB.

### Patient-Facing Dashboard and Tasks

Study participants can manage individual study activities on their smartphone via ehive’s patient-facing dashboard. This user-friendly interface provides an overview of the study tasks and activities as well as a list of completed tasks and future tasks. Participants can also access a summary of the types of device data they are sharing with the study team. Additional features include detailed information about the study’s IRB approval, contact information of the study team, and the signed consent form. Participants can withdraw from the study through the app.

Through the task list on the ehive dashboard, participants can complete custom or standardized surveys as well as view videos and educational content. Additional integration with the EHR is possible. Participants can grant permission for the EHR to be read via Apple’s HealthKit. When connected, EHR data are pulled directly from the HealthKit database into the ehive platform via Fast Healthcare Interoperability Resources (FHIR). Currently, this interface allows participants to visualize the data collected from connected devices or scored survey results. However, in the future, it is planned to enable the visualization of wearable metrics by participants within the patient-facing dashboard. Depending on the specific study, this information can be masked to minimize bias.

The ehive app is designed to collect multimodal data, including PROMs. PROM questionnaires are delivered as study tasks, with participants’ responses saved directly in the ehive database in the FHIR Questionnaire Response format. New questionnaires are created using a configuration file that is deployed to the ehive server and added to a study’s task schedule. Questionnaires are created, scheduled, modified, and deployed without requiring an app update. The ehive app has built-in support for 23 question types (ie, multiple select, year, yes or no select, drag or drop reorder, text, etc) and branching logic to show or hide questions based on the participant’s responses. The ehive backend is integrated with the REDCap (Research Electronic Data Capture; Vanderbilt University) platform, enabling it to write and read questionnaire responses with REDCap, if requested by the study team. Previously used surveys are stored in a data library to allow for ease of use in future studies.

### Data Integration

The ehive platform is integrated with external data sources using two primary mechanisms: (1) via a web application programming interface (API) or (2) via a software development kit (SDK). For security purposes, ehive integrates only with web APIs that implement the Open Authorization 2.0 (OAuth 2.0) protocol. OAuth 2.0 is an authorization standard that enables a third party to request a service on behalf of the resource owner. Using the OAuth 2.0 protocol, the resource owner retains full control of an individual’s log-in credentials and the shared data. When initiating a request to share data, ehive specifies exactly which scopes (data types) the study is interested in. The participant chooses which of these scopes he or she wishes to authorize, and then the external service releases a short-lived access token and a refresh token. The access token grants temporary access to the authorized data. After the access token expires, a new access token may be released using the provided refresh token. These tokens are stored in the database and used for server-to-server communication, allowing ehive to pull external data without continued interaction with the ehive mobile app. The study participant can revoke ehive’s access at any time, whereupon the refresh token will be invalid, no new access tokens will be released, and no more data will be obtained from the participant.

OAuth 2.0-enabled web APIs are ubiquitous among commercial and research-grade wearable device companies. As the authorization logic is identical among external services and all API methods are simple http requests, little effort is required to integrate with a new data source. In addition, web APIs can be used to integrate data for any service that chooses to expose an API (eg, device data, genetic data, and social media data). SDKs are primarily used to integrate wearable devices. Unlike web APIs that permit server-to-server communication, SDKs require specific code to be bundled with the ehive mobile app to handle communication with the device. Using an SDK is significantly more complicated than using a web API. There are few similarities among SDKs, even among those from the same manufacturer (eg, HealthKit and SensorKit [Apple Inc]), making meaningful abstraction impossible. Furthermore, using SDKs can lead to imbalances between platforms because some SDKs are only available on iOS (eg, HealthKit), whereas others are only available on Android (eg, Samsung Health [Samsung Electronics]). Additionally, iOS and Android require the use of different programming languages and tools. Therefore, when an SDK is to be made available on iOS and Android devices, it must be implemented separately for each platform. These complexities prolong the time required to integrate a new SDK into platforms such as ehive. In addition to the primary integration mechanisms discussed earlier, ehive has access to unique data set integrations only available within the MSHS, such as genomic and proteomic biobanks. This data integration is automated and does not require any action on the part of participants.

Multi–data sets such as pathology data, radiology images, EHR in Observational Medical Outcomes Partnership format, and genetics data are stored in a different environment within the MSHS called AIR.MS for efficiency and security purposes [19]. They can be accessed and integrated with ehive data with IRB approval and participant consent. The ehive platform collects information such as medical record numbers, name, sex, date of birth, and zip code. When the bootstrapping process is initiated in both environments, a lookup table with the hash value of the medical record number, the date of birth, and a random value is exchanged between the 2 domains using a public or private key exchange protocol. After the bootstrapping process, when a new patient is added to AIR.MS, a unique hash value is inserted into the AIR.MS lookup table. The same steps are performed on ehive when a new participant signs in to assess whether he or she is in MSHS. A new record is added to the ehive lookup table. If the security of the lookup table is compromised, a new public or private key is generated, and a random value is shared. Both AIR.MS and ehive must then recompute their lookup tables for future matching between the data sets. Principal investigators (PIs) interested in using multiple data sets from both environments must be granted access and have IRB approval. Once access is granted, a software agent returns the number of data points available for the time range requested by the PI. The PI can then verify the data quantities returned and decide whether they meet the criteria to build the cohort. If the cohort meets the PI criteria, an automatic process deploys a new virtual machine accessible to the researchers on the IRB who have valid Mount Sinai Azure Active Directory credentials. The virtual machine has automated access only to the data requested by the PI.

### Data Infrastructure

**Figure 6:**
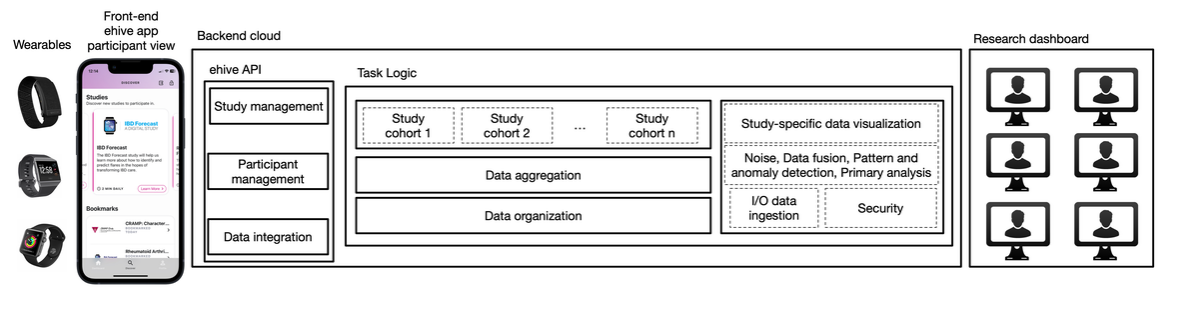
The ehive platform encompasses a front-end patient-facing smartphone app, backend cloud, and research dashboard. External to the ehive digital ecosystem are third-party apps and wearable devices. Their integration to ehive is mainly through the REST application programming interface paradigm. When data integration through the REST application programming interface is unavailable, as with Apple Watch, ehive accesses the data through software development kits. The ehive data architecture is based on Azure cloud, managed by the Mount Sinai Health System. Investigators interact with the collected data, the study’s status, and the study’s configuration through the research portal that runs on the Azure cloud. IO: input-output.

The ehive backend runs on Health Insurance Portability and Accountability Act–compliant Azure cloud infrastructure (Figure 6). It uses Docker (Docker Inc) and a microservice architecture pattern to simplify the maintenance and deployment of new features. Each service has its database, which contains the data required to perform its duties. No sensitive information is stored within the microservices themselves. Database access keys and API keys are stored in Azure Key Vault, and all data are stored in an encrypted Postgres database. A flexible Postgres service that scales dynamically to meet the current workload is used to improve performance and availability. App-related data are stored primarily using relational database techniques. However, PROMs and data from external integrations are stored directly in JavaScript Object Notation format. Using JavaScript Object Notation, the ehive mobile app and backend can communicate using the FHIR standard. This design choice allows ehive data to be interpretable by other health organizations, a benefit of which is the possibility of generating interoperable data analyses and merging with EHR and other health data sources.

Data shared with ehive are authorized on a per-study basis. If a participant is enrolled in multiple studies that request the same data integration, the participant will be asked to provide authorization for each study individually. This process provides transparency and control, allowing participants to decide which data are accessed by each study. Gaps in wearable device data can occur for several reasons. For example, this can occur (1) when the device runs out of battery and is unable to sync with the app’s backend, (2) when the participant ceases wearing the device, or (3) when a data sync fails for unforeseen technical reasons. It is vital to the success of each study to minimize gaps in the collected data. Several services run on the backend of ehive to check whether new data are available and whether any gaps exist. In case of a sync failure, the backend will make ad hoc data requests to fill in any missing data. When the gaps cannot be filled via ad hoc requests, an automated message is delivered to participants, notifying them of the issues with their device use patterns.

## Results

### The ehive Platform

The ehive platform was launched in 2020 and has since hosted 8 ongoing or completed studies, with 7 additional studies planned for launch. This study composition is 6 observational cohort studies and 2 clinical trials. The observational studies encompass a range of disease and health states. These include studies recruiting healthy health care workers throughout the MSHS for the development of algorithms to predict COVID-19 infections, studies for understanding the psychological well-being of health care workers during the COVID-19 pandemic, and studies aimed at developing machine learning algorithms to predict psychological states from passively collected wearable data. Beyond the MSHS, the platform has hosted 2 national studies enrolling individuals with chronic diseases from across the United States aimed at predicting disease exacerbations using wearable device data [20,21]. The clinical trials hosted on ehive are fully digital. The Warrior Shield study was such a siteless trial, which enrolled 127 health care workers and aimed at building resilience using digital interventions that modified heart rate variability. The physiological effects of the intervention were monitored using wearable devices.

### Participant Recruitment and Engagement

**Figure 7:**
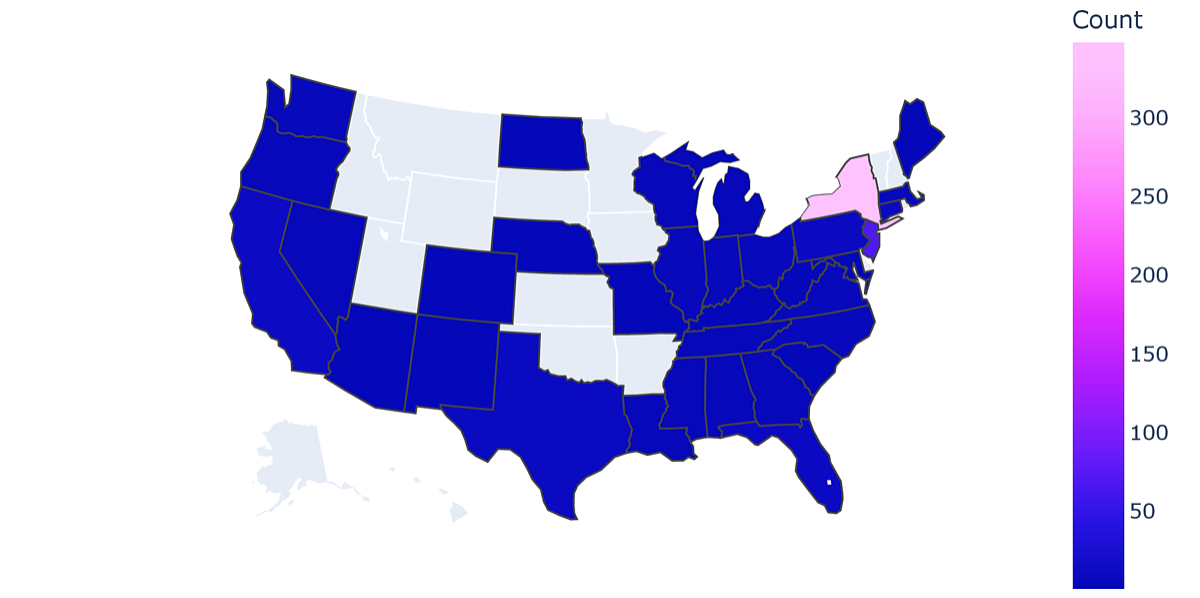
The number of individuals participating in ehive-related studies in each state in the United States.

Through January 2023, a total of 1484 participants downloaded the ehive app, with 1044 (70.35%) of them meeting the inclusion criteria for study enrollment. Over 51 million wearable device–based data points have been collected and over 132,241 surveys have been conducted through the ehive app. This demonstrates ehive’s ability to recruit across multiple studies and the digital ecosystem’s ability to simultaneously run multiple individual cohort studies and clinical trials. Importantly, ehive-based studies have engaged with and recruited diverse populations. For example, the Warrior Watch study, which developed machine learning algorithms aimed at predicting COVID-19 infections, enrolled 297 participants across racial and ethnic groups (Table 1) [22]. Although most ehive study participants are centered around the New York City metropolitan area, individuals across 36 states within the United States have enrolled (Figure 7).

**Figure 8:**
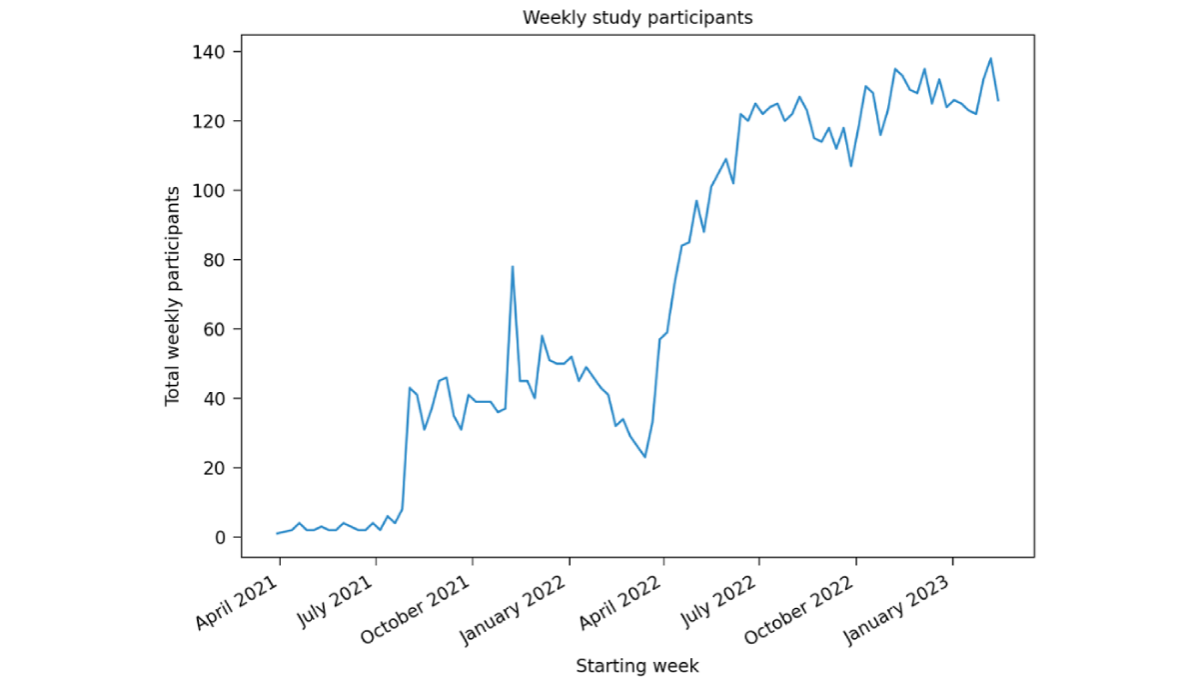
A trend line of the total weekly number of ehive participants from April 2021 through January 2023, demonstrating high rates of use and engagement.

**Figure 9:**
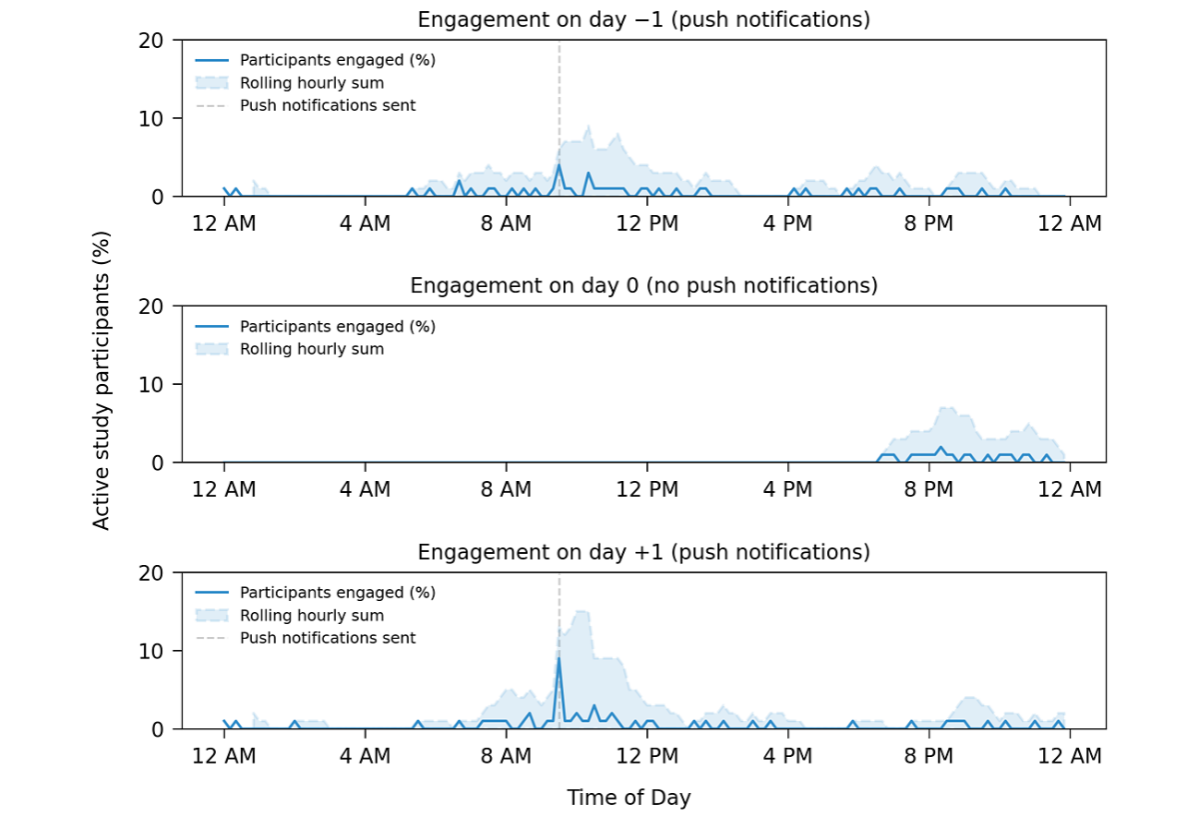
An example of engagement in the ehive app on 3 sequential days. Push notifications were delivered to study participants on day −1 and day +1 as a reminder to complete daily survey tasks. No notifications were delivered on day 0. The percentage of participants who engaged with ehive and the rolling hourly sum of participants using the app is visualized over each 24-hour period.

Participant engagement and the use of the ehive platform have steadily increased as the number of studies supported by the app have increased. In addition, modifications that enhance engagement and retention have been continually made and improved, as discussed earlier. The app has an average of 148 (SD 11.43) unique daily users over a 7-day period (April 2023; Figure 8). Highlighting the role that light-touch engagement measures play in the interaction with ehive, we found that measures such as push notifications result in a marked increase in user engagement shortly after being implemented (Figure 9). This highlights the ability of these light-touch measures to remind and engage participants in ongoing studies that require either daily or sporadic tasks within the app.

**Table 1:** The ehive platform has enrolled diverse patient populations. The demographic profile of the participants enrolled in one ehive study, the Warrior Watch Study, is described to highlight participant diversity (n=297).

Characteristics		Value
Age (years), mean (SD)		36.3 (9.8)
Sex (female), n (%)		204 (68.7)
**Race, n (%)**		
	Asian	73 (24.6)
	Black	29 (9.8)
	White	108 (36.4)
	Other	43 (14.5)
Hispanic ethnicity, n (%)		44 (14.8)

## Discussion

### Principal Findings

There are limited descriptions of centralized digital research applications in the literature to help guide their development. The ehive app represents such a model that can be used to grow digital health research. Here, we provide an overview of the infrastructure and design of ehive, which was developed to help mitigate many of the limitations that have historically impacted digital studies. This platform has successfully hosted 8 observational studies and clinical trials that include over 1044 participants across 36 states. Such a centralized digital ecosystem fosters the opportunity for conducting digital health research at scale across multiple studies, participant populations, and data modalities.

The advent of the COVID-19 pandemic has highlighted the need to be able to perform research remotely and engage traditionally underrepresented patient populations. The ehive platform represents a model that academic and other institutions can use to meet these needs and grow research opportunities in an increasingly remote and digital world. The modifiability of platforms such as ehive enables multiple types of study that incorporate various data streams to be conducted. This includes opportunities for the digitization of clinical trials, which has been increasingly emphasized by industry and funding agencies, including the National Institute of Health [6,23]. Digital solutions present an opportunity to streamline clinical trial costs, improve efficiency through investigator support, provide novel physiological data streams, and offer new end points. In addition and as demonstrated, they enable expanded study recruitment, compliance monitoring, and enhanced automated retention modalities [24].

Such platforms enable the integration of multimodal data streams, including wearable outputs, biobank specimens (ie, genomic and proteomic biobanks), EHR outputs, and PROMs. The integration of multiple data streams allows data sources to complement each other, amplifying the derived information beyond each individual data stream [25]. The ability of centralized digital research platforms to add multiple data modalities that can evaluate information on different scales results in improved predictive ability and the potential for artificial intelligence and machine learning techniques to unlock novel capabilities. Although this is implemented at scale in large biobanks, such as the United Kingdom biobank or the National Institutes of Health All of Us initiative, centralized digital platforms, such as ehive, enable this performance on a modifiable and tailored basis [26,27]. An additional benefit of a central research app is that capital improvements in the app are translated across studies, thereby optimizing institutional or organizational investment in digital research infrastructure. This allows the entire digital ecosystem to benefit from system upgrades and modifications and avoids the duplication of effort and, thereby, costs that would occur if similar improvements were applied to single-use apps.

However, only limited descriptions of such platforms are available in the literature. Published descriptions of modifiable central digital research platforms focus on specific study types. For example, the StudyU platform is a customizable app that enables clinicians and researchers to design and implement N-of-1 trials. This unique platform is similar to ehive to the extent that it leverages its centralized features to host individual studies that capitalize on central app developments [28]. However, descriptions of fully modifiable centralized platforms that can support multiple studies and study types are lacking. In academics, such platforms include the myPHD app from Stanford University, whereas in industry, the evidations research app and Apple Research app (Apple Inc) have similar capabilities [11,29,30]. However, robust descriptions of their capabilities have not been published.

The modifiability of the ehive research ecosystem enables its evolution and expansion over time. Several future app developments are planned. Given the importance of participant compliance and engagement, ehive updates are focused on maximizing this aspect of the app. A micropayment system is planned, which will allow participants to easily track their study progress and acquire points or monetary compensation after hitting study milestones. This feature will enable the automatic payment of participants through ehive, mitigating the need for study coordinators to track and remit compensation. In addition, an expansion of the patient-facing dashboard is planned. This will enable participants to not only track their wearable data in aggregate but also download their granular wearable data during the study period. This will increase participant engagement with the app, enable participants to share their data with their doctors if needed, and convey the impression that the collected data are being engaged with and used by the researchers. Further scaling of the ehive platform through research collaborations with academic and industry partners as well as through the exploration of platform licensing options is also planned.

A strength of the ehive platform is the ability of individual studies to use existing features or develop new ones. However, this app has several important limitations. Although we have worked to develop light-touch measures to maintain engagement with the app, we still note that participant engagement decreases over time. Although this is a limitation in all studies, ongoing efforts are needed to continually improve how participants interact with the app. Our planned micropayment system and an improved patient-facing dashboard will hopefully further engage participants. Another limitation in studies hosted on ehive is restrictions on data access. Wearable device companies often have limitations on the type and granularity of data that can be accessed. In addition, there can be intellectual property limitations on the downstream uses of these data, which can impact the ownership of the study results and algorithms. This highlights the need for greater collaboration between device companies and health researchers as well as solutions in which raw data access can be obtained for reasonable compensation. An additional limitation is that if study participants do not have automatic updates turned on, the ehive team has to manually reach out to participants to have them update the app. Although this happens infrequently if there is a major app update that impacts a large number of participants, it can be a significant limitation. Finally, there are significant costs related to running and maintaining a centralized research platform that require upfront institutional investment. Although subsequent app-driven revenue streams can support ongoing operations, the initial development costs often require external support.

### Conclusions

The ehive app is a promising proof-of-concept centralized research solution for complex health care systems that see value in building a secure mobile health research platform that can support multiple studies. It is a novel tool that offers investigators the capability to expand their research beyond their institution, engage in large-scale longitudinal studies, and combine multimodal data streams. It serves as a model for groups seeking to develop similar digital health research programs.

## Abbreviations

API: application programming interface
DDP: Digital Discovery Program
EHR: electronic health record
FHIR: Fast Healthcare Interoperability Resources
HPI.MS: Hasso Plattner Institute for Digital Health at Mount Sinai
IRB: institutional review board
MSHS: Mount Sinai Health System
PI: principal investigator
PROM: patient-reported outcomes measure
REDCap: Research Electronic Data Capture
SDK: software development kit

## Data Availability

The data sets generated and analyzed during this study are not publicly available. This is because the data summaries presented represent the composites of individual datasets from multiple studies. Each study has its own restrictions on data access.

## References

[1] Goodday SM, Karlin E, Alfarano A, Brooks A, Chapman C, Desille R, Rangwala S, Karlin DR, Emami H, Woods NF, Boch A, Foschini L, Wildman M, Cormack F, Taptiklis N, Pratap A, Ghassemi M, Goldenberg A, Nagaraj S, Walsh E, Friend S (2021). An alternative to the light touch digital health remote study: the stress and recovery in frontline COVID-19 health care workers study. JMIR Form Res.

[2] Vogels EA (2020). About one-in-five Americans use a smart watch or fitness tracker. Pew Research Center.

[3] Snyder M, Zhou W (2019). Big data and health. Lancet Digit Health.

[4] Steinhubl SR, Muse ED, Topol EJ (2015). The emerging field of mobile health. Sci Transl Med.

[5] Lee J-A, Choi M, Lee SA, Jiang N (2018). Effective behavioral intervention strategies using mobile health applications for chronic disease management: a systematic review. BMC Med Inform Decis Mak.

[6] Steinhubl SR, Wolff-Hughes DL, Nilsen W, Iturriaga E, Califf RM (2019). Digital clinical trials: creating a vision for the future. NPJ Digit Med.

[7] Dobkin BH, Dorsch A (2011). The promise of mHealth: daily activity monitoring and outcome assessments by wearable sensors. Neurorehabil Neural Repair.

[8] McConnell MV, Shcherbina A, Pavlovic A, Homburger JR, Goldfeder RL, Waggot D, Cho MK, Rosenberger ME, Haskell WL, Myers J, Champagne MA, Mignot E, Landray M, Tarassenko L, Harrington RA, Yeung AC, Ashley EA (2017). Feasibility of obtaining measures of lifestyle from a smartphone app: the MyHeart counts cardiovascular health study. JAMA Cardiol.

[9] Nguyen NH, Martinez I, Atreja A, Sitapati AM, Sandborn WJ, Ohno-Machado L, Singh S (2022). Digital health technologies for remote monitoring and management of inflammatory bowel disease: a systematic review. Am J Gastroenterol.

[10] Meyerowitz-Katz G, Ravi S, Arnolda L, Feng X, Maberly G, Astell-Burt T (2020). Rates of attrition and dropout in app-based interventions for chronic disease: systematic review and meta-analysis. J Med Internet Res.

[11] How evidation works. Evidation.

[12] Simblett S, Greer B, Matcham F, Curtis H, Polhemus A, Ferrão J, Gamble P, Wykes T (2018). Barriers to and facilitators of engagement with remote measurement technology for managing health: systematic review and content analysis of findings. J Med Internet Res.

[13] Facts and figures. Mount Sinai.

[14] (2019). Mount Sinai and Hasso plattner institute launch new institute for digital health. Mount Sinai.

[15] Digital discovery program. Hasso Plattner Institute for Digital Health.

[16] McDonald AM, Knight RC, Campbell MK, Entwistle VA, Grant AM, Cook JA, Elbourne DR, Francis D, Garcia J, Roberts I, Snowdon C (2006). What influences recruitment to randomised controlled trials? A review of trials funded by two UK funding agencies. Trials.

[17] Frampton GK, Shepherd J, Pickett K, Griffiths G, Wyatt JC (2020). Digital tools for the recruitment and retention of participants in randomised controlled trials: a systematic map. Trials.

[18] Pratap A, Neto EC, Snyder P, Stepnowsky C, Elhadad N, Grant D, Mohebbi MH, Mooney S, Suver C, Wilbanks J, Mangravite L, Heagerty PJ, Areán P, Omberg L (2020). Indicators of retention in remote digital health studies: a cross-study evaluation of 100,000 participants. NPJ Digit Med.

[19] AIR·MS – AI Ready Mount Sinai. Mount Sinai.

[20] IBD forecast. Hasso Plattner Institute for Digital Health at Mount Sinai.

[21] Rheumatoid arthritis forecast. Hasso Plattner Institute for Digital Health at Mount Sinai.

[22] Hirten RP, Danieletto M, Tomalin L, Choi KH, Zweig M, Golden E, Kaur S, Helmus D, Biello A, Pyzik R, Charney A, Miotto R, Glicksberg BS, Levin M, Nabeel I, Aberg J, Reich D, Charney D, Bottinger EP, Keefer L, Suarez-Farinas M, Nadkarni GN, Fayad ZA (2021). Use of physiological data from a wearable device to identify SARS-CoV-2 infection and symptoms and predict COVID-19 diagnosis: observational study. J Med Internet Res.

[23] (2019). Digital clinical trials workshop: creating a vision for the future. National Institutes of Health.

[24] Inan OT, Tenaerts P, Prindiville SA, Reynolds HR, Dizon DS, Cooper-Arnold K, Turakhia M, Pletcher MJ, Preston KL, Krumholz HM, Marlin BM, Mandl KD, Klasnja P, Spring B, Iturriaga E, Campo R, Desvigne-Nickens P, Rosenberg Y, Steinhubl SR, Califf RM (2020). Digitizing clinical trials. NPJ Digit Med.

[25] Boehm KM, Khosravi P, Vanguri R, Gao J, Shah SP (2022). Harnessing multimodal data integration to advance precision oncology. Nat Rev Cancer.

[26] Denny JC, Rutter JL, Goldstein DB, Philippakis A, Smoller JW, Jenkins G, Dishman E, All of Us Research Program Investigators (2019). The "All of Us" research program. N Engl J Med.

[27] Bycroft C, Freeman C, Petkova D, Band G, Elliott LT, Sharp K, Motyer A, Vukcevic D, Delaneau O, O'Connell J, Cortes A, Welsh S, Young A, Effingham M, McVean G, Leslie S, Allen N, Donnelly P, Marchini J (2018). The UK Biobank resource with deep phenotyping and genomic data. Nature.

[28] Konigorski S, Wernicke S, Slosarek T, Zenner AM, Strelow N, Ruether DF, Henschel F, Manaswini M, Pottbäcker F, Edelman JA, Owoyele B, Danieletto M, Golden E, Zweig M, Nadkarni GN, Böttinger E (2022). StudyU: a platform for designing and conducting innovative digital N-of-1 trials. J Med Internet Res.

[29] MyPHD homepage. My Personal Health Dashboard.

[30] Apple research. Apple Store.

